# Tension Pneumatocele due to *Enterobacter gergoviae* Pneumonia: A Case Report

**DOI:** 10.1155/2012/808630

**Published:** 2012-09-29

**Authors:** Emeka B. Kesieme, Chinenye N. Kesieme, George O. Akpede, Kelechi E. Okonta, Andrew E. Dongo, Adewuyi M. Gbolagade, Sylvester U. Eluehike

**Affiliations:** ^1^Department of Surgery, Irrua Specialist Teaching Hospital, PMB 8, Irrua, Edo State, Nigeria; ^2^Department of Paediatrics, Irrua Specialist Teaching Hospital, PMB 8, Irrua, Edo State, Nigeria; ^3^Department of Surgery, University College Hospital, Ibadan, Nigeria; ^4^Department of Medical Microbiology, Irrua Specialist Teaching Hospital, PMB 8, Irrua, Edo State, Nigeria; ^5^Department of Radiology, Irrua Specialist Teaching Hospital, PMB 8, Irrua, Edo State, Nigeria

## Abstract

Pneumatocele formation is a known complication of pneumonia. Very rarely, they may increase markedly in size, causing cardiorespiratory compromise. Many organisms have been implicated in the pathogenesis of this disease; however, this is the first report of tension pneumatocele resulting from *Enterobacter gergoviae* pneumonia. We report a case of a 3-month-old Nigerian male child who developed two massive tension pneumatoceles while on treatment for postpneumonic empyema due to *Enterobacter gergoviae* pneumonia. Tube thoracostomy directed into both pneumatocele resulted in complete resolution and recovery. *Enterobacter gergoviae* is a relevant human pathogen, capable of causing complicated pneumonia with fatal outcome if not properly managed. In developing countries where state-of the-art radiological facilities and expertise for prompt thoracic intervention are lacking, there is still room for nonoperative management of tension pneumatocele especially in very ill children.

## 1. Introduction

Pneumatoceles are thin walled air-filled pulmonary cysts which develop as a complication of pneumonia. They can also result secondary to trauma and positive pressure ventilation.

Postpneumonic pneumatocele may result from rupture of bronchiolar walls due to necrosis causing formation of air corridors and subsequent accumulation of air between the visceral pleura and parenchyma resulting in subpleural pneumatocele [1]. Expanding pneumatocele may result from a check-valve action at the site of rupture [2].

Spontaneous resolution of pneumatocele usually occurs after a few weeks alongside the pneumonic process; however in rare cases, it is complicated by dramatic presentation of tension with cardiorespiratory compromise.

To the best of our knowledge, this is the first report implicating *Enterobacter gergoviae* pneumonia as a cause of tension pneumatocele.

## 2. Case Presentation

We report the case of a 3-month-old male who presented with cough, catarrh, difficulty in breathing, and high-grade, continuous fever of one week duration. There was no history of contact with someone with chronic cough. The child was being exclusively breastfed. On examination at presentation, he was ill looking, mildly pale, dyspneic, not cyanosed, and not dehydrated. The weight for age and other anthropometric parameters were normal. Vital signs measurement revealed a heart rate of 180 beats/min, respiratory rate of 52 breaths/min, and a temperature of 37.8°C. Examination revealed widespread rhonchi in both lung fields and no crepitation was heard. An initial diagnosis of bronchopneumonia to rule out bronchiolitis was made. Initial full blood count done revealed packed cell volume of 25%, white cell count of 13,700/mm^3^ with relative neutrophilia (65%), and absolute platelet count of 617,000/mm^3^. Blood film for malaria was negative and Mantoux test done was negative (5 mm area of induration). A sample for blood culture was taken at the peak of pyrexia, and the patient was placed on intravenous antibiotics, (iv ampicillin/sulbactam 50 mg/kg/dose 8 hourly subsequently changed to iv cefuroxime 120 mg/kg/day 8 hourly and iv gentamicin 5 mg/kg/day), intravenous infusion, oxygen by nasal prongs, and intermittent salbutamol nebulization.

Patient did not however improve as the vital signs worsened after 3 days. Respiratory rate increased to 74 breaths/min. Chest Radiograph showed loss of costophrenic and cardiophrenic angles and homogenous opacification of the left lung.

**Figure 1:**
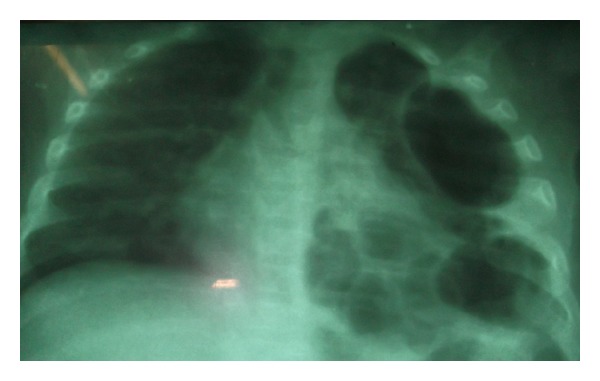
Repeat chest radiographs revealed 2 huge pneumatoceles and several smaller ones on the left lung zone.

Patient could not be managed in the intensive care unit (ICU) because there is no functional paediatric ICU. Arterial blood gases estimation machine is also not available. Left tube thoracostomy was inserted into the left pleural space. It drained purulent effluent which was subsequently taken for microscopy, culture and sensitivity, and acid fast bacilli. Seven days after thoracostomy, repeat chest radiograph revealed 2 huge pneumatoceles and several smaller ones (Figure 1). A chest computerized tomography scan machine is not available at this center, and the patient was unable to afford one at the nearest center. 

The result of the pleural fluid microscopy and blood culture yielded heavy growth of *Enterobacter gergoviae* sensitive to ciprofloxacin and gentamicin. The organism was isolated on sheep blood agar and MacConkey agar plates. The antibiotic sensitivity test is by modified Kirby-bauer disk diffusion method.

The patient's condition continued to deteriorate. His respiratory rate became 80 breaths/min and pulse rate 187 beats/min, The antibiotic regimen was changed from iv cefuroxime to iv ceftazidime 100 mg/kg/day and iv gentamicin 5 mg/kg/day was continued. There was marked improvement 36 hours after commencing ceftazidime. 

**Figure 2:**
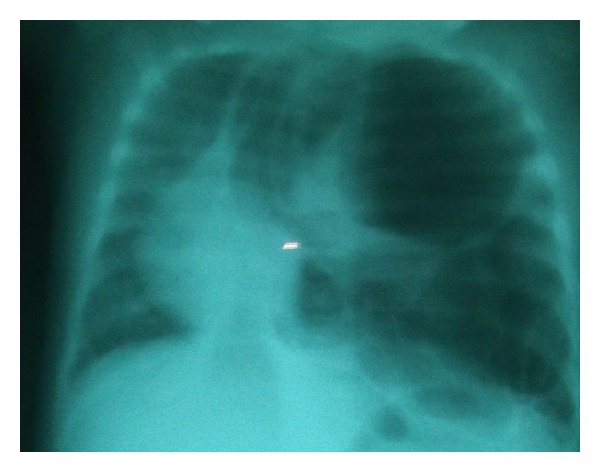
Repeat chest radiograph revealed enlargement of the pneumatoceles causing massive mediastinal shift to the right.

**Figure 3:**
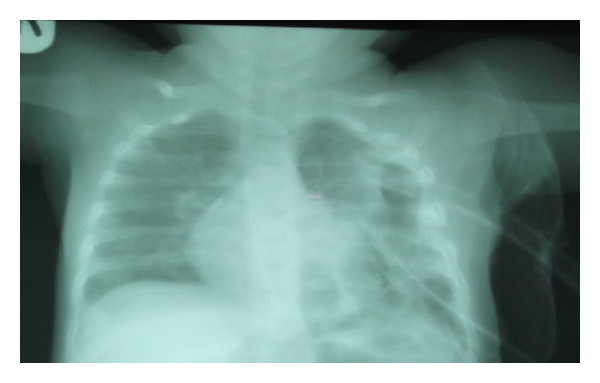
Double thoracostomy tube drains were inserted into each pneumatocele separately.

**Figure 4:**
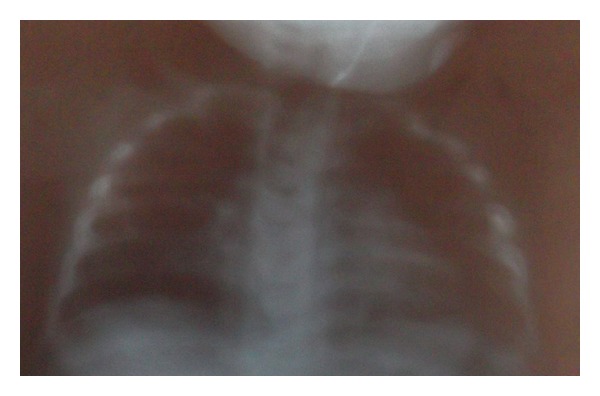
Chest Radiograph done a month after discharge.

A repeat chest radiograph revealed enlargement of the pneumatoceles causing massive mediastinal shift to the right (Figure 2). Double thoracostomy tube drains were inserted into each pneumatocele separately (Figure 3). There was a massive air escape into the underwater seal drain. The patient's condition continued to improve, and he was discharged after a month on admission on syrup ciprofloxacin 7.5 mg/kg/dose 12 hrly for one week. Chest radiograph done just before discharge revealed no obvious lung pathology. Subsequent clinical and radiologic evaluation in the outpatient clinic revealed no abnormality (Figure 4).

## 3. Discussion 

Tension pneumatocele can be defined as expanding air-filled pulmonary cyst, usually of postinfectious origin, compressing adjacent area of the lung and resulting in cardiorespiratory compromise.

Most pneumatoceles occur as a complication of pneumonia. They are known to resolve spontaneously over several weeks or months. Rarely, they may result in complications of tension, infection, and rupture which may be life threatening and requires prompt attention. Tension pneumatocele enlarges significantly compressing adjacent lung and mediastinum resulting in cardiovascular collapse.

Most of the cases reported in the literature were infants and children. Apart from the index patient who presented with double tension pneumatoceles at 3 months of age, only one patient reported by Papageorgiou et al. developed clinical and radiologic features of traumatic pneumatocele on the 42nd day of life [3]. Most of the cases of adult patients reported in the literature had an additional underlying pathology [4].

Our patient had multiple pneumatoceles and two of them were under tension, all confined to the left lung. Multiple tension pneumatoceles are very rare in the pediatric population. There is no consensus in the literature regarding which lung is mostly affected.

Staphylococcus aureus is the most commonly implicated organism responsible for pneumatocele formation; however, other reports have implicated pneumonia caused by *Streptococcus pneumoniae*, *Haemophilus influenzae*, *E. coli*, *S. pyogenes*, *Serratia marcescens*, *K. pneumoniae*, adenovirus, *M. tuberculosis*, *Pneumocystis jiroveci*, *Pneumocystic carinii*, and *Acinetobacter calcoaceticus* [5, 6]. 

*Enterobacter gergoviae* belongs to the family Enterobacteriaceae, and it is a relatively rare human pathogen. However, it has been implicated as a cause of bacteremia in a neonatal ICU in Johor, Malaysia [7], lower respiratory tract infection [8], and traumatic endophthalmitis [9]. 

A pneumatocele may occur in the absence of significant empyema, although our patient developed tension pneumatocele in a background of postpneumonic empyema.

Many modalities of treatment have been described in the literature. Image-guided percutaneous drainage, compression, catheter drainage and tube drainage are effective treatment modalities for single tension pneumatocele. Although we succeeded with tube thoracostomy drainage of the double left-sided tension pneumatocele, Wu and Chen reported failed thoracostomy drainage in a patient with multiple pneumatoceles [10]. Pneumonostomy and lung resection surgery (lobectomy and pneumonectomy) have been performed in patients with multiple tension pneumatoceles or after failure of tube thoracostomy drainage. High-frequency oscillatory ventilation has been used to treat tension pneumatocele in a patient with severe pneumonia, tension pneumatocele, and pneumothorax receiving conventional mechanical ventilation (CMV) [11]. 

## 4. Conclusion

This report represents an important contribution in establishing *Enterobacter gergoviae* as a cause of tension pneumatocele. In a resource-limited center like ours, there is a role for tube thoracostomy in the management of tension pneumatocele; however, if they do not resolve or if they are more than 2, lung resection surgery becomes the preferred modality of management.
